# Establishment and interspecific associations in two species of *Ichthyocotylurus *(Trematoda) parasites in perch (*Perca fluviatilis*)

**DOI:** 10.1186/1756-3305-4-85

**Published:** 2011-05-20

**Authors:** Anna Faltýnková, Anssi Karvonen, E Tellervo Valtonen

**Affiliations:** 1 Department of Biological and Environmental Science, University of Jyväskylä, PO Box 35 (YA), FI-40014 Jyväskylä, Finland; 2 Department of Biological and Environmental Science, Centre of Excellence in Evolutionary Research, P.O. Box 35, FI-40014 University of Jyväskylä, Finland; 3 Institute of Parasitology, Biology Centre of the Czech Academy of Sciences, Branišovská 31, 370 05 České Budějovice, Czech Republic

## Abstract

**Background:**

Co-infections of multiple parasite species in hosts may lead to interspecific associations and subsequently shape the structure of a parasite community. However, few studies have focused on these associations in highly abundant parasite species or, in particular, investigated how the associations develop with time in hosts exposed to co-infecting parasite species for the first time. We investigated metacercarial establishment and interspecific associations in the trematodes *Ichthyocotylurus variegatus *and *I. pileatus *co-infecting three age cohorts of young perch (*Perca fluviatilis*).

**Results:**

We found that the timing of transmission of the two *Ichthyocotylurus *species was very similar, but they showed differences in metacercarial development essentially so that the metacercariae of *I. pileatus *became encapsulated faster. Correlations between the abundances of the species were significantly positive after the first summer of host life and also within the main site of infection, the swim bladder. High or low abundances of both parasite species were also more frequent in the same host individuals than expected by chance, independently of host age or size. However, the highest abundances of the species were nevertheless observed in different host individuals and this pattern was consistent in all age cohorts.

**Conclusions:**

The results suggest similar temporal patterns of transmission, non-random establishment, and facilitative rather than competitive associations between the parasite species independently of the age of the infracommunities. However, we suggest that spatial differences in exposure are most likely responsible for the segregation of the parasite species observed in the few most heavily infected hosts. Regardless of the underlying mechanism, the result suggests that between-species associations should be interpreted with caution along with detailed examination of the parasite distribution among host individuals.

## Background

Co-infections, i.e. infections of hosts by multiple parasite species are the rule in host-parasite interactions in nature [1-4]. These infections may lead to different types of intraspecific and interspecific associations, which may shape the structure of a parasite community (reviewed in [5]). Associations take place within individual hosts (infracommunity), and may cause numerical (changes in numbers of one species) and/or functional (shifts in species distribution) responses in the co-infecting species [5]. One important aspect of these associations is the initial parasite establishment, the pattern and sequence of which may determine the magnitude and direction of associations in a parasite species-pair. For example, recent evidence has shown that the sequence of parasite establishment and host immunisation may significantly change the outcome of associations between co-infecting parasite species [4]. However, few studies have investigated parasite establishment in naïve infracommunities, where co-infecting parasite species establish in the same host individuals for the first time. Such investigations could reveal how the associations develop with host age and increasing parasite abundances, particularly in species accumulating in hosts with time. In the present study, we investigated the establishment and interspecific associations of two trematode species, *Ichthyocotylurus pileatus *and *I. variegatus*, co-infecting young age cohorts of their second intermediate host, perch (*Perca fluviatilis*).

*Ichthyocotylurus *species have complex life cycles, which include snail (*Valvata *spp.), fish and bird hosts [6-9]. Metacercariae infect a wide range of freshwater fish species [10,11] and accumulate in fish with age, resulting in high abundances that may reach hundreds or even thousands of parasites in an individual fish [6,7,12-14]. Indeed, numerically *Ichthyocotylurus *spp. are among the most abundant parasite species of freshwater fish. Four species of *Ichthyocotylurus *are known to infect fish in Europe [6,7] and their taxonomic identity has been verified with molecular techniques [15]. Patterns of infection in fish, such as seasonality of infections and age-intensity profiles, are also reasonably well known [6,7,12,16-18]. Recently, cercarial characteristics including quantitative temporal patterns in cercarial emergence from the snail hosts have been explored [19,20]. On the other hand, dynamics of co-infections in these species are not well known, but form an interesting area of research as high infection intensities of these species could lead to a range of interspecific and intraspecific associations within the hosts. This would be particularly likely in species-pairs infecting the same host organs. Furthermore, these parasites encapsulate in host tissues after establishment and can remain infective to the definitive host for a considerable length of time [6,7]. However, very little is known about the dynamics of development and encapsulation in these parasites, especially in systems where two species co-infect the host at the same time.

The aim of this paper was to investigate establishment and interspecific associations between *I. pileatus *and *I. variegatus *metacercariae in wild perch (*Perca fluviatilis*). By repeatedly sampling three young age cohorts of perch (0, 1 and 2 years of age), we recorded metacercarial establishment and development in naïve and developing infracommunities. Second, we analysed interspecific associations between the parasite species separately in each age cohort and recorded numerical responses in parasite abundances. This allowed an insight into development of associations within the infracommunities during the progression of parasite accumulation. We also investigated possible functional responses by determining how the species were distributed between the two sites of infection in fish (swim bladder and kidney) and if there were numerical responses within the sites.

## Materials and methods

Sampling of the perch was carried out in Lake Konnevesi (62° N 26° E), a large oligotrophic lake in Central Finland with a surface area of 113 km^2^, mean depth of 13 m, and the maximum depth of 56 m. Fish were captured with fish traps and seine nets monthly from May to October 2007 from one area at the lake (see [8]). A total of 499 perch belonging to three age cohorts were examined: 0+ fish (n = 218, mean length ± SE = 50.5 ± 1.2 mm) hatched in early summer 2007, 1+ fish (n = 121, mean length = 84.4 ± 1.0 mm) hatched in the previous summer, and 2+ fish (n = 160, mean length = 115.5 ± 1.4 mm) hatched two years ago. The cohorts were clearly distinguishable according to the size of the fish. The older cohorts were sampled monthly in May-August (1+) and May-October (2+). The youngest cohort (0+) was sampled monthly in July-October so that additional samples were taken biweekly in July and September. Fish were brought fresh to the laboratory, where they were killed, and measured for length and weight. Their internal organs were compressed between two glass plates and examined for metacercariae of *Ichthyocotylurus *under a stereomicroscope. Infections of *I. pileatus *and *I. variegatus *were observed only in swim bladder and kidney.

**Figure 1:**
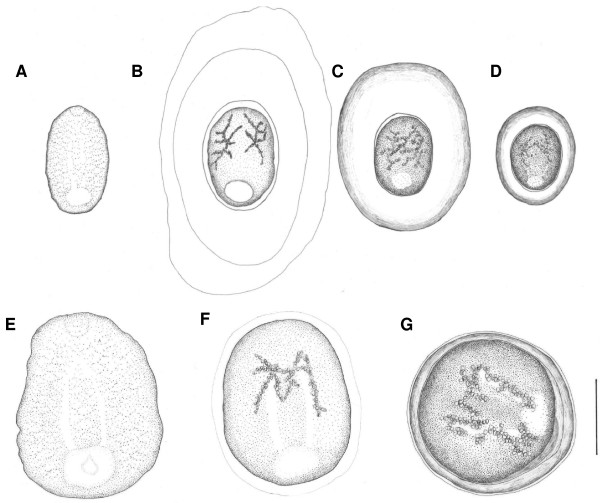
**Developmental stages of the metacercariae of *Ichthyocotylurus pileatus *and *I. variegatus *in perch**. **A-D ***I. pileatus*; **A **- recently established metacercaria, **B-C **- encapsulating metacercaria, **D **- fully developed and encapsulated metacercaria. **E-G ***I. variegatus*; **E **- recently established metacercaria, **F **- encapsulating metacercaria, **G **- fully developed and encapsulated metacercaria. Morphology of the metacercariae and cysts are based on averages from 40 individuals (B-C, D, G) or on measurements from few individuals taken under a light microscope (A, E, F). The scale bar is 300 μm.

The structure of the parasite cysts and the internal morphology of the metacercariae necessary for species identification and separation were examined under a light microscope. Measurements (in micrometres) were taken from live specimens. Further routine identification and counting was carried out with aid of a stereomicroscope. The species identification was based on the internal metacercarial morphology described in keys in [6,7,9,11]. Metacercariae of both species were classified into three distinct categories according to the stage of development: (i) recently established, without a cyst, (ii) encapsulating with a visible formation of the cyst, and (iii) fully developed and encapsulated metacercariae (Figure 1). All developmental categories of both species were observed in swim bladder and kidney. Exact measurements were taken from encapsulating and fully developed metacercariae of *I. pileatus*, and fully developed metacercariae of *I. variegatus*, from which representative numbers of good-quality specimens could be obtained. The other developmental stages were either too rare (see results) or too fragile for representative measurements. In these cases, measurements were taken from few individuals and descriptive graphical illustrations were produced with aid of a drawing attachment of a light microscope.

### Statistical analyses

Data on the establishment of *I. variegatus *and *I. pileatus *were analysed using MANOVA separately for the age groups of fish. Data were log (n+1) transformed to meet the assumptions of the analysis when necessary. MANOVAs were followed by ANOVAs separately on the abundances of the species. Interspecific associations between the parasite species were analysed from data pooled within the age groups (see below) using Pearson correlation analysis. As the abundance of the parasite species depended on the size of the fish, residuals from length-abundance regressions were extracted for all age groups of fish and used in the correlation analyses to control for the effect of fish size. Correlations were also used to analyse associations between the species within the different host organs, i.e. swim bladder and kidney. All correlations were run by excluding fish individuals from which both parasite species were absent (i.e. double-zeros).

## Results

### Parasite establishment in fish

**Figure 2:**
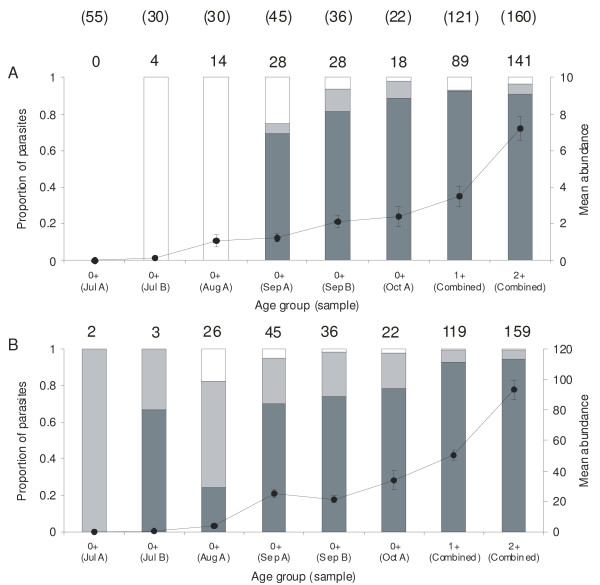
**Proportions of the three developmental stages of the metacercariae of *Ichthyocotylurus variegatus *(A) and *I. pileatus *(B) in three age cohorts of perch (0+, 1+ and 2+) in Lake Konnevesi, Central Finland: recently established metacercariae (white bars), encapsulating metacercariae (light grey bars) and fully developed, encapsulated metacercariae (dark grey bars)**. Solid line with dots presents mean parasite abundance (± SE). Numbers on top in parentheses indicate total sample size for each month; numbers on top of each column indicate number of infected fish. Note that the data are presented separately for the sampling times in 0+ fish; data for 1+ and 2+ fish have been combined for the sampling times. Data for *I. pileatus *in 0+ fish are the same presented in [20].

First metacercariae were detected in 0+ fish in July after which the prevalences of both parasite species increased steadily, reaching 100% for *I. pileatus *in September and 81.8% for *I. variegatus *in October (Table 1). Prevalence of co-infections followed a similar pattern. Prevalences were high throughout the monthly samples in 1+ and 2+ fish (Table 1). The abundance of the parasite species in 0+ fish also increased with time (MANOVA on log-transformed data: F_10,424 _= 35.361, p < 0.001; ANOVA on *I. variegatus*: F_5,212 _= 25.947, p < 0.001; ANOVA on *I. pileatus*: F_5,212 _= 216.016, p < 0.001) and reached the highest values in October being 2.41 ± 0.55 for *I. variegatus *and 33.91 ± 6.45 for *I. pileatus *(Figure 2). The increase was most pronounced in July-August, but there was no longer any change in abundance of *I. pileatus *in September-October (MANOVA on log-transformed data: F_4,200 _= 2.582, p = 0.038; ANOVA on *I. pileatus*: F_2,100 _= 1.365, p = 0.260). In *I. variegatus*, there was a difference in the abundance in September-October (ANOVA: F_2,100 _= 3.735, p = 0.027), but this was no longer significant in pair-wise comparisons (Tukey multiple comparisons: p > 0.05 for all). The abundances continued to increase in 1+ and 2+ fish (Figure 2), but there was no difference in abundance of the species between the sampling times in 1+ fish (MANOVA: F_6,234 _= 1.793, p = 0.101). In the 2+ fish, the analysis indicated a difference in the abundance of *I. variegatus *between the sampling times (MANOVA: F_8,310 _= 1.978, p = 0.049; ANOVA on *I. variegatus*: F_4,155 _= 3.233, p = 0.014), but, again, this was no longer significant in pair-wise comparisons (Tukey multiple comparisons: p > 0.05 for all). There was no difference in the abundance of *I. pileatus *between the sampling times in 2+ fish (ANOVA: F_4,155 _= 0.558, p = 0.694).

**Table 1:** Prevalence of *I. variegatus *and *I. pileatus*, and co-infections of both species in monthly samples of three age cohorts of perch caught from Lake Konnevesi, Central Finland

Age	Month	n	*I. variegatus*	*I. pileatus*	Co-infection
0+	July A	55	0.0	3.6	0.0
	July B	30	13.3	10.0	6.7
	August	30	46.7	86.7	43.4
	September A	45	62.2	100.0	62.2
	September B	36	77.8	100.0	77.8
	October	22	81.8	100.0	81.8
	All	218	42.4	61.5	40.8

					
1+	May	30	70.0	96.7	70.0
	June	31	80.6	100.0	80.6
	July	30	63.3	96.7	60.0
	August	30	80.0	100.0	80.0
	All	121	73.6	98.3	72.7

					
2+	May	40	92.5	100.0	92.5
	June	30	83.3	96.7	83.3
	July	30	76.7	100.0	76.7
	August	30	96.7	100.0	96.7
	September	30	93.3	100.0	93.3
	All	160	88.8	99.4	88.8

Sampling of 0+ aged fish was done twice in July and September.

### Interspecific differences in morphology and development of the metacercariae

Recently established metacercariae differed in size so that those of *I. pileatus *were substantially smaller than *I. variegatus*, and this difference remained throughout the metacercarial development (Figure 1). The size ratio in fully developed metacercariae was approximately 1:1.8 (mean length of *I*. *pileatus *= 373 μm, *I*. *variegatus *= 677 μm, Table 2). Arrangement of the cyst wall was also different between the species. In encapsulating *I. pileatus*, a thick concentric layer was formed around the hyaline layer, whereas in *I. variegatus *this layer was thin and inconspicuous. Similarly, the cyst wall of fully developed metacercariae of *I. pileatus *consisted of two clearly separate layers whereas in *I. variegatus *the layers were compact. Thickness of the wall was also greater in encapsulating *I. pileatus*, representing approximately 60-100% of the body length of the metacercariae. In fully developed metacercariae, however, the wall became significantly thinner representing only 20% of the metacercarial body length. The corresponding thickness in the fully developed *I. variegatus *was 10%. Moreover, fully developed metacercariae of *I. pileatus *and *I. variegatus *differed in body size and shape (oval *vs*. spherical) and in sensitivity to rupture of the cyst under mechanical pressure (difficult *vs*. easy).

**Table 2:** Dimensions (in micrometers) of metacercariae of *I. pileatus *and *I. variegatus *recovered from perch

Species, stage of development	Whole metacercaria		Body of metacercaria (without cyst wall)	
**N = 40**	**range (min-max)**	**mean ± SD**	**range**	**mean ± SD**

*I. pileatus*, encysting metacercariae	496-1008 × 448-816	660 ± 93 × 566 ± 83	237-424 × 179-301	310 ± 39 × 236 ± 31
*I. pileatus*, fully encysted	288-536 × 237-473	373 ± 51 × 315 ±55	224-314 × 173-365	261 ± 22 × 211 ± 31
*I. variegatus*, fully encysted	544-977 × 344-767	677 ± 98 × 620 ± 84	432-819 × 320-640	567 ± 92 × 508 ± 68

There was also a difference in the occurrence of different developmental stages between the species so that the proportion of encapsulating metacercariae in 0+ fish was consistently higher in *I. pileatus *over the sampling times (paired-samples t-test on arcsine-transformed data: t_4 _= 2.976, p = 0.041; Figure 2). Also, the recently established metacercarial stages of *I. pileatus *occurred rarely from late July onwards during the highest parasite transmission and were virtually absent compared to *I. variegatus *although this was not significant at five percent level when all sampling times were included (paired-samples t-test on arcsine-transformed data: t_4 _= 2.020, p = 0.114; Figure 2). In October, the proportions of the recently established metacercarial stages were similar between the parasite species (paired-samples t-test on arcsine-transformed data: t_17 _= 0.587, p = 0.565), and remained roughly constant thereafter in 1+ and 2+ fish (Figure 2).

### Interspecific associations

**Figure 3:**
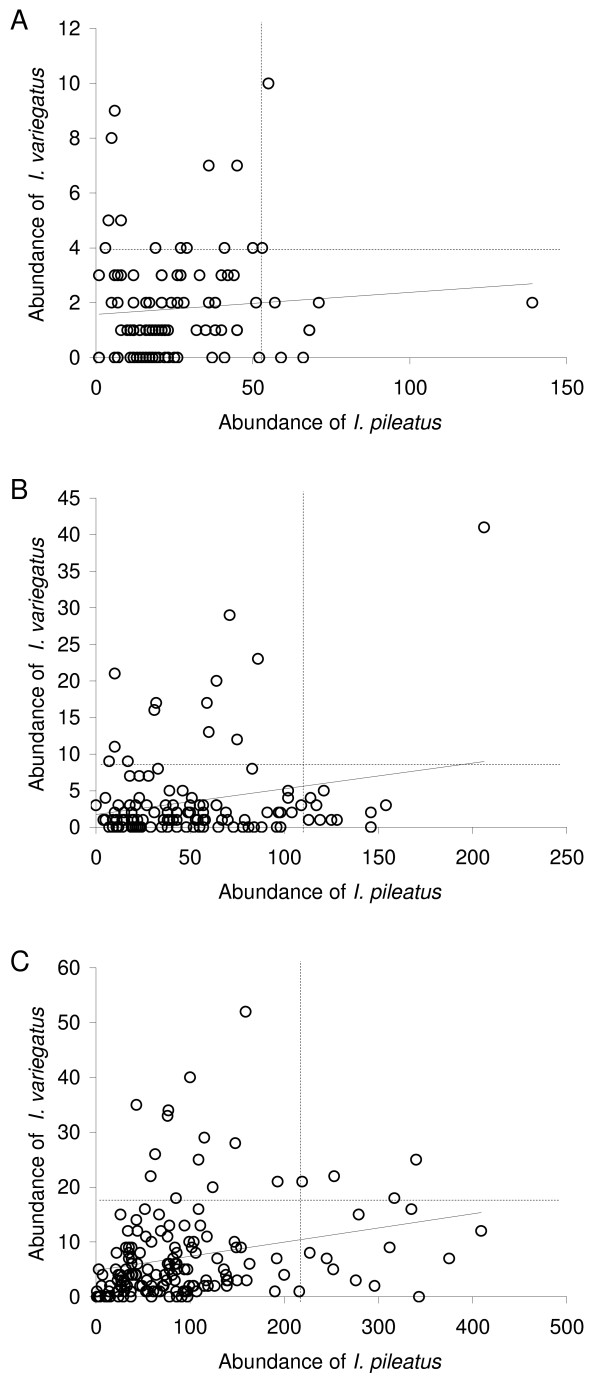
**Relationships between abundance of *Ichthyocotylurus variegatus *and *I. pileatus *in three age cohorts of perch, 0+ (A) n = 103, 1+ (B) n = 120 and 2+ (C) n = 159**. Solid lines represent linear regressions indicating the direction of the relationship. Dashed lines in each panel indicate 10% of the fish individuals most heavily infected with *I. variegatus *(horizontal line) and 10% most heavily infected with *I. pileatus *(vertical line).

Interspecific associations between *I. variegatus *and *I. pileatus *were analysed by pooling the data from all sampling times within each age group of fish (the last three sampling times in 0+ fish, and all data in 1+ and 2+ fish). This was done as the previous analyses indicated no difference in parasite abundance between these sampling times (see above). Association between the abundance of the parasite species became significantly positive with fish age. While no such relationship was detected in 0+ fish (Pearson correlation on residuals from length-abundance regressions: r = 0.073, n = 103, p = 0.461; Figure 3a), the relationship was significant in 1+ fish (r = 0.222, n = 120, p = 0.015) although in these fish the pattern was strongly influenced by one data point (Figure 3b). The relationship was significantly positive also in 2+ fish (r = 0.240, n = 159, p = 0.002; Figure 3c). Moreover, the residual numbers of the metacercariae of both parasite species, taken from the length-abundance relationships, had the same sign, positive or negative, in 67/103, 71/120 and 93/159 cases in 0+, 1+ and 2+ fish, respectively. This occurred more frequently than expected by chance in all three age cohorts [χ^2 ^= 9.330, df = 1, p = 0.002 (0+); χ^2 ^= 4.033, df = 1, p = 0.045 (1+); χ^2 ^= 4.585, df = 1, p = 0.032 (2+)]. The result indicates that fish individuals infected with more (or less) metacercariae of *I. variegatus *than expected based on their size, harboured also more (or less) metacercariae of *I. pileatus*.

However, the highest abundances of both parasite species still occurred in different fish individuals. This became evident when we extracted 10% of the fish individuals most heavily infected with *I. variegatus*, and 10% most heavily infected with *I. pileatus *from the data, and analysed differences in parasite abundances between these groups separately for the species (Figure 3). If there were no segregation to different hosts, the abundances should not differ. However, this null hypothesis was refuted as the abundances of both parasite species were significantly different between the groups in all age cohorts (Table 3). On average, abundances of *I. variegatus *in the most heavily infected 10% of the fish were 3.0 times higher compared to abundances of *I. variegatus *in the 10% most heavily infected with *I. pileatus*. The corresponding number for *I. pileatus *was 2.4.

**Table 3:** Mean abundance (±SE) of *I. variegatus *and *I. pileatus *in three age cohorts of perch using data from 10% of the fish individuals most heavily infected with each of the parasite species

			Most heavily infected 10%			Paired-samples t-test		
**Cohort**	**Parasite**	**n**	***I. variegatus***	***I. pileatus***	**Ratio**	**t**	**d.f**.	**P**

0+	*I. variegatus*	10	6.3 ± 0.7	2.2 ± 1.0	2.86	3.029	9	0.014
	*I. pileatus*	10	25.3 ± 6.9	68.8 ± 8.1	2.72	5.413	9	<0.001
1+	*I. variegatus*	12	19.1 ± 2.6	5.4 ± 3.3	3.52	6.396	11	<0.001
	*I. pileatus*	12	59.3 ± 15.5	133.2 ± 7.8	2.25	7.358	11	<0.001
2+	*I. variegatus*	16	28.2 ± 2.2	10.7 ± 2.0	2.64	6.108	15	<0.001
	*I. pileatus*	16	135.1 ± 20.3	293.4 ± 14.4	2.17	5.711	15	<0.001

The ratio indicates proportional difference between the abundances. Significant differences in abundances of both parasite species within each cohort indicate that the highest abundances of the species occurred in different fish individuals.

The majority of the individuals of both species were found in the swim bladder, but the proportion of *I. pileatus *[73.9 ± 0.01%, data combined for 0+ (September-October), 1+ and 2+ fish] was lower compared to *I. variegatus *(87.7 ± 0.01%; paired-samples t-test on arcsine transformed data: t_303 _= 12.960, p < 0.001). However, correlations between the abundances of the species in the swim bladder were positive [Pearson correlation: r = 0.083, n = 103, p = 0.405 (0+ fish); r = 0.243, n = 120, p = 0.007 (1+ fish); r = 0.209, n = 159, p = 0.008 (2+ fish)] and the proportion of *I. pileatus *in swim bladder did not depend on the abundance of *I. variegatus *[Pearson correlation: r = 0.058, n = 103, p = 0.560 (0+ fish); r = 0.120, n = 119, p = 0.194 (1+ fish); r = 0.087, n = 159, p = 0.275 (2+ fish)]. This suggests that there was no competitive exclusion between the species in this site.

## Discussion

Co-infections of parasite species in a host may lead to interspecific associations, which can shape the overall community structure [5]. These associations are particularly likely in systems where numerically abundant parasite species infect the same location in a host. In the present paper, we explored the establishment and interspecific associations in two abundant trematode species, *Ichthyocotylurus variegatus *and *I. pileatus*, co-infecting the same organs in their second intermediate fish host, *Perca fluviatilis*. By sampling young cohorts of fish, we investigated how parasites established and co-occurred in naïve 0+ fish which were exposed to the parasites for the first time, as well as in 1+ and 2+ fish that harboured infections also from the previous years. Parasite transmission to the youngest cohort indicated that the seasonality of transmission was very similar between the species. The first metacercariae appeared in fish in July, which corresponds to the timing of infections in the first intermediate snail hosts (*Valvata macrostoma*) [8]. Afterwards, abundances of both species increased steadily, but the abundance of *I. pileatus *was consistently higher compared *I. variegatus *suggesting interspecific differences in transmission dynamics. This concurs with earlier findings reporting lower abundances in *I. variegatus *compared with the other *Ichthyocotylurus *species [6,17]. One possible factor underlying these differences is the spatial heterogeneity in transmission between the species. In our earlier study [8], we examined trematode infections in *Valvata *snails in the same littoral habitats (depth <6 m) where the fish were sampled in the present investigation. However, we did not find infections of *I. variegatus *from those snails in any of the monthly samples [8], which suggests that the parasite transmission takes place elsewhere. For example, it is possible that *I. variegatus *is transmitted to perch in deeper areas of the lake where specific conditions such as lower water temperature could result in lower rate of transmission to fish. Such a small-scale interspecific segregation of the transmission is surprising given that both parasite species mature in birds (see discussion in [8]), which effectively disseminate parasite eggs to the environment. Nevertheless, the co-occurrence of different developmental stages in the 0+ fish suggests that the fish, caught from one location, move actively between these infection 'hotspots' [21] and become exposed to both parasite species roughly at the same time.

We observed that the recently established metacercariae of *I. variegatus *were proportionally more common compared to *I. pileatus*, while the same was true for the encapsulating stages of *I. pileatus*. This suggests that metacercariae of *I. pileatus *become more rapidly encapsulated after establishment, but also that the time between encapsulation and maturation in metacercariae of *I. variegatus *is relatively short. Exact reasons for these developmental differences between the species are unknown, but they may include processes such as the magnitude of host immune responses. For example, the capsules surrounding metacercariae of *I. pileatus *were consistently thicker compared to *I. variegatus*, which suggests stronger host responses against that species. This is somewhat surprising given the significantly larger size of *I. variegatus*, which intuitively would require more resources taken from the host. However, experimental work is needed to address the different hypothetical scenarios related to interactions between the rate of encapsulation, metacercarial development, and magnitude of parasite-induced damage to the host. Detailed description of the metacercarial development and encapsulation can also be used as a complementary tool in morphological identification of the species, which has previously been based solely on the morphometrics of the fully developed metacercariae. For example, the notable interspecific size difference of the metacercariae already in the early stages of development suggests that examination of the internal morphology is not necessarily needed for separation of these species. Moreover, differences in the diameter and shape of the cyst wall (oval in *I. pileatus *and round in *I. variegatus*) provide further means for separation of the species.

We also analysed interspecific associations between the species and investigated how these develop with time and accumulation of the metacercariae in hosts of different age. We found that the species were positively associated and this pattern was emphasised in the older age cohorts. Similarly, both parasite species primarily infected the swim bladder and the associations within this site were also positive. Taken together, these results suggest facilitative rather than competitive associations between the species, possibly emerging from the overlapping temporal transmission dynamics (see above) and common interests in transmission to avian definitive hosts [6,7,11,12,18]. Similar positive associations have recently been described, for example, in monogeneans infecting marine and freshwater fish [22-24] and trematodes infecting eyes of fish [4]. Thus, our results corroborate with this line of evidence reporting non-competitive but also non-random community structure.

However, the highest parasite abundances nevertheless tended to occur in different host individuals and this pattern was consistent throughout the age cohorts of fish (see also [25]). In other words, the most heavily infected 10% of the fish harboured parasites mainly from one of the species. Mechanisms underlying such a pattern of infection are unclear, but direct competitive interactions between the species seem unlikely. This is because (i) we observed mainly positive associations in lower abundances, (ii) both species mainly infected the same organ in the host, and (iii) the metacercariae are relatively inactive after encapsulation making direct interspecific interactions unlikely. It is also unlikely that fish individuals most susceptible to infection from one of the parasite species would be among the most resistant to the other species (but see below), especially given the positive association in infection in lower abundances. Moreover, these young infracommunities should be far from saturation as the parasite numbers continue to increase in older age classes and may reach even thousands per individual fish [26]. However, it could be that these differences emerge as a result of spatial heterogeneity in exposure to the parasite species (see above). The risk of infection in trematodes is not spatially uniform [21,27-29] and similar interspecific heterogeneities in spatial exposure exist also in the present system [8]. Under such circumstances, it is possible that individuals occupying different areas in a lake become exposed to infective stages of different parasite species. Such an unequal or sequential exposure to one species may also lead to responses in the host that will influence the community structure later when the host becomes co-exposed to other parasite species [4]. In the present study, all fish were caught from the same specific location, which indicates that habitats of the fish were at least partly overlapping, although this does not exclude the possibility of past heterogeneities in exposure. Regardless of the underlying mechanism, however, this pattern of infection suggests that analyses conducted on different sub-sets of the host population may lead to different interpretations of the nature of the associations between parasite species.

## Conclusions

To conclude, development of *I. variegatus *and *I. pileatus *metacercariae in perch, as indicated by the proportional occurrence of different developmental stages and their morphology, was different despite the similarities in timing of the transmission. Positive associations between the species and their occurrence in the same organs despite high parasite abundances support facilitative interspecific associations in this system. However, this result was partly based on correlative analyses, which do not necessarily account for heterogeneities in the distribution of parasites in a host population. For example, the fact that the highest parasite abundances were occurring in different host individuals was evident only when we divided the population into sub-sets and conducted separate analyses. We emphasise that correlative analyses exploring interspecific interactions should be conducted along with a detailed examination of the distribution of parasites among individual hosts.

## Competing interests

The authors declare that they have no competing interests.

## Authors' contributions

AF carried out the sampling, processing, identification and description of the material, and AK conducted the statistical analyses. All authors planned the study together, participated in writing of the manuscript, read and approved the final draft.
